# Effect of 2-Cys Peroxiredoxins Inhibition on Redox Modifications of Bull Sperm Proteins

**DOI:** 10.3390/ijms222312888

**Published:** 2021-11-28

**Authors:** Agnieszka Mostek-Majewska, Anna Janta, Anna Majewska, Andrzej Ciereszko

**Affiliations:** Institute of Animal Reproduction and Food Research of Polish Academy of Sciences, 10-748 Olsztyn, Poland; a.janta@pan.olsztyn.pl (A.J.); a.majewska@pan.olsztyn.pl (A.M.); a.ciereszko@pan.olsztyn.pl (A.C.)

**Keywords:** peroxiredoxins, bull sperm, redox proteomics, oxidative post-translational modifications of proteins

## Abstract

Sperm peroxiredoxins (PRDXs) are moonlighting proteins which, in addition to their antioxidant activity, also act as redox signal transducers through PRDX-induced oxidative post-translational modifications of proteins (oxPTMs). Despite extensive knowledge on the antioxidant activity of PRDXs, the mechanisms related to PRDX-mediated oxPTMs are poorly understood. The present study aimed to investigate the effect of bull sperm 2-Cys PRDX inhibition by Conoidin A on changes in oxPTM levels under control and oxidative stress conditions. The results showed that a group of sperm mitochondrial (LDHAL6B, CS, ACO2, SDHA, ACAPM) and actin cytoskeleton proteins (CAPZB, ALDOA, CCIN) is oxidized due to the action of 2-Cys PRDXs under control conditions. In turn, under oxidative stress conditions, 2-Cys PRDX activity seems to be focused on antioxidant function protecting glycolytic, TCA pathway, and respiratory chain enzymes; chaperones; and sperm axonemal tubulins from oxidative damage. Interestingly, the inhibition of PRDX resulted in oxidation of a group of rate-limiting glycolytic proteins, which is known to trigger the switching of glucose metabolism from glycolysis to pentose phosphate pathway (PPP). The obtained results are expected to broaden the knowledge of the potential role of bull sperm 2-Cys in both redox signal transmission and antioxidant activity.

## 1. Introduction

Peroxiredoxins (PRDXs) are well-conserved, highly expressed thiol-dependent enzymes forming up to 1% of the cellular protein content present in all known species [1,2]. Over the last decade, numerous studies have pointed out the prominent role of PRDXs as highly efficient scavengers of hydrogen peroxide (H_2_O_2_), thus regulating the antioxidant defense system [3,4]. It has been widely proven that PRDXs can also serve as highly sensitive sensors of H_2_O_2_ and play a pivotal role in redox-mediated pathways by oxidizing specific redox-sensitive proteins throughout signal transduction from H_2_O_2_. Moreover, PRDX oligomers formed by overoxidation of PRDXs are considered molecular chaperones, similar to heat shock proteins [5,6].

There are two possible mechanisms by which oxidative equivalents can be transferred from PRDXs to their target proteins. The first mechanism concerns the reaction of sulfenic acid of oxidized PRDX with the cysteine (Cys) thiol of the partner protein, followed by thiol–disulfide exchange. The second mechanism involves the reaction between the thiol of the partner protein with the disulfide of PRDX through the typical thiol–disulfide exchange mechanism [7]. Oxidation of intra-cellular thiols leads to alterations of redox-sensitive target protein conformation, ligand binding, enzymatic activity, and protein–protein interactions [8]. Breakthrough research on this subject [9] has shown that PRDX2 acts as a H_2_O_2_ signal transmitter, enabling redox regulation of one of the transcription factors—STAT3. Furthermore, it was shown that ”cytokine-induced STAT3 signaling is accompanied by PRDX2 and STAT3 oxidation, and is regulated by PRDX2 expression levels”.

Peroxiredoxins can be classified into three groups, according to the number and localization of conserved Cys residues: typical 2-Cys PRDXs (isoforms 1–4), atypical 2-Cys PRDX (PRDX5), and 1-Cys PRDX (PRDX6). Typical 2-Cys PRDXs function as obligate homodimers. Each 2-Cys PRDX monomer contains two reactive cysteines: peroxidatic (Cys_p_) and resolving (Cys_R_). Typical 2-Cys PRDXs scavenge H_2_O_2_ (as well as peroxynitrite and tert-butyl hydroperoxide) through the formation of a sulfenic acid (Cys-SOH) on the Cys_p_*,* with the subsequent formation of a disulfide bond with Cys_R_ by the second monomer. Exceptionally, PRDX5 contains Cys_p_ and Cys_R_ on the same monomer, forming an internal disulfide during the reaction cycle. In turn, 1-Cys PRDX (PRDX 6) contains only Cys_p_, and requires a separate redox-relay binding partner such as glutathione [10,11]. Cys-SOH formed during the scavenging of H_2_O_2_ leads to the inactivation of PRDXs. The reactivation mechanism of inactive 2-Cys PRDXs is based on a rapid disulfide reduction to -SH by thioredoxin (Trx) and the Trx reductase system using NADPH as an electron donor, whereas, in the case of PRDX6, a sulfenic acid group is reduced by the glutathione-GSH-transferase system [2,12].

Human PRDXs are regulated by a variety of post-translational modifications (PTMs), including acetylation, ubiquitination, glutathionylation, phosphorylation, and tyrosine nitration. A total of 26 modified residues have been identified amongst the six different human isoforms: 10 for PRDX1, five for PRDX2, two for PRDX3, and five for PRDX6 [13]. However, the most abundant PTMs concern different types of oxidation—such as disulfide formation, sulfinylation, sulfonylation, S-nitrosylation, and glutathionylation—that modulate the activity of PRDXs and contribute to the regulation of cell signaling.

A unique feature of peroxiredoxins is the ability to change their structure and function depending on the concentration of H_2_O_2_. Under low H_2_O_2_ concentrations, a dynamic equilibrium between PRDXs dimers and decamers exists. In turn, under high H_2_O_2_ concentrations, the decamers stack to form high-molecular-weight (HMW) oligomers, leading to a loss of peroxidative activity and gaining the function of a molecular chaperone [14]. This interesting alteration of the function and structure of PRDXs is most likely to protect cells from oxidation-induced protein unfolding. To recover the original peroxidative activity, overoxidized PRDXs are repaired by sulfiredoxin in an ATP-dependent manner. Due to their remarkable sensitivity to fluctuating H_2_O_2_ concentrations, PRDXs act as molecular switches influenced by changes in peroxide levels, whether for stress or non-stress related signaling [15].

Over the past few years, peroxiredoxins have been associated with male reproduction, especially spermatozoa. They play a critical role in the regulation of sperm function and male fertility and are considered essential elements in the antioxidant defense of spermatozoa [8,16]. It has been reported that insufficient PRDX activity affects sperm motility, capacitation, mitochondrial membrane potential, and DNA integrity [3,16,17]. Thus far, the localization of PRDXs in the testis, epididymis, and mature spermatozoa has been described [3,18,19,20]. Recent research also demonstrated that PRDXs may regulate protein tyrosine phosphorylation in spermatozoa via a PKA-dependent mechanism [20]. Spermatozoa from infertile men contain lower amounts of PRDX 1 and PRDX 6, accompanied by increased levels of sperm lipid peroxidation, DNA damage, and impairment of sperm motility. Furthermore, inhibition of PRDX6 in mouse spermatozoa resulted in oxidative stress and a reduced ability to remove lipid peroxides [21]. The information gathered so far indicates an extremely important role of PRDXs in the functioning of mammalian sperm, thus creating new therapeutic routes for the improvement of sperm quality. However, despite the existing knowledge on the subject of structures, functions, and PTMs of PRDXs reviewed by [13,14,15], a clear understanding of their role in physiology—especially in redox signaling—remains elusive. Further development of redox proteomics techniques will enable the discovery of the complete redox proteome as well as the identification of proteins and pathways modulated by PRDX in physiological processes.

Although PRDXs have been described in detail, in terms of their antioxidant activity in sperm [11,20,22], little is known about their equally important function of redox signaling. Recent studies have shown that PRDXs play a primary role in redox signaling by assimilating oxidative equivalents from H_2_O_2_ and transferring them selectively to target groups of (redox-responsive) proteins [7]. Moreover, PRDXs seem to be the main players that accomplish redox regulation in capacitating spermatozoa [23], which has been confirmed by studies showing that the inhibition of PRDX activity prevents sperm capacitation [16]. The aim of this research was to verify the effect of PRDX inhibition with Conoidin A (Con A) on the parameters of sperm motility, viability, ROS+ cells, and the level of reversible oxPTMs of sperm proteins under control conditions and oxidative stress. The obtained results are expected to broaden the knowledge of the redox modifications of proteins in bull sperm controlled by 2-Cys PRDXs. We decided to use Con A in this study, as it is a potent inhibitor of all types of 2-Cys PRDXs (isoforms 1–4) [11,20] and has been reported to inhibit 2-Cys PRDX activity through covalent binding to catalytic cysteines [24]. With such an approach, we aimed to unravel the role of PRDXs in sperm physiology and to identify the target proteins of PRDX-mediated redox signaling.

## 2. Results

### 2.1. Sperm Motility

**Figure 1:**
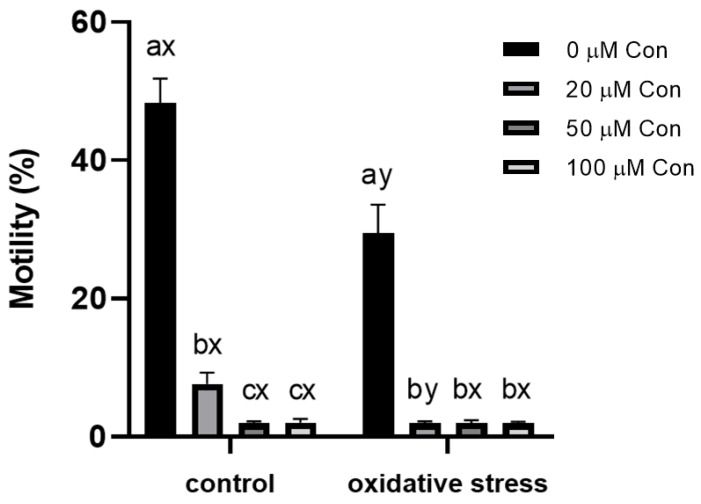
Effect of different concentrations of Con A on percentage of motile bull spermatozoa (%) under control conditions (without menadione) and oxidative stress conditions (100 µM menadione). a, b, c: differences within control or oxidative stress between different concentrations of Con A. x, y: differences within a given concentration of Con A between control or oxidative stress (*n* = 6). Data are expressed as the mean ± SEM; significance, *p* < 0.05.

CASA measurement of sperm motility revealed that this parameter was greatly affected by Con A. At 20 µM of Con A, the population of motile sperm decreased from 48.3 ± 3.2% to 7.6 ± 1.5%, and at higher concentrations, the motility was suppressed almost completely (to 2.0 ± 0.5%). Subjecting the sperm to oxidative stress mediated by menadione inhibited the motility of spermatozoa to 29.4 ± 3.8%, and with the addition of even the lowest concentration of Con A (20 µM), the motility was almost completely suppressed (reaching 1.9 ± 0.2%; see Figure 1).

### 2.2. Sperm Viability

**Figure 2:**
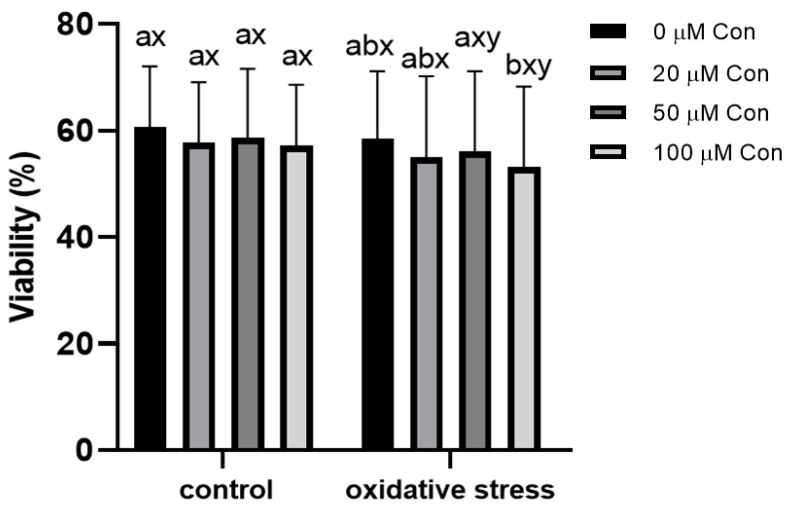
Effect of different concentrations of Con A on percentage of viable bull spermatozoa (%) under control conditions (without menadione) and oxidative stress conditions (100 µM menadione). a, b: differences within control or oxidative stress between different concentrations of Con A. x, y: differences within a given concentration of Con A between control or oxidative stress (*n* = 6). Data are expressed as mean ± SEM; significance, *p* < 0.05.

The viability of sperm was 60.71 ± 10.68% in non-treated samples and was not significantly affected by the applied Con A concentrations. The menadione-induced oxidative stress also did not change the percentage of viable sperm. Under oxidative stress, the sperm viability decreased with the highest concentration of Con A (100 µM), where viability decreased from 58.5 ± 11.84% to 53.2 ± 14.15% (Figure 2).

### 2.3. ROS-Positive Cell Content

**Figure 3:**
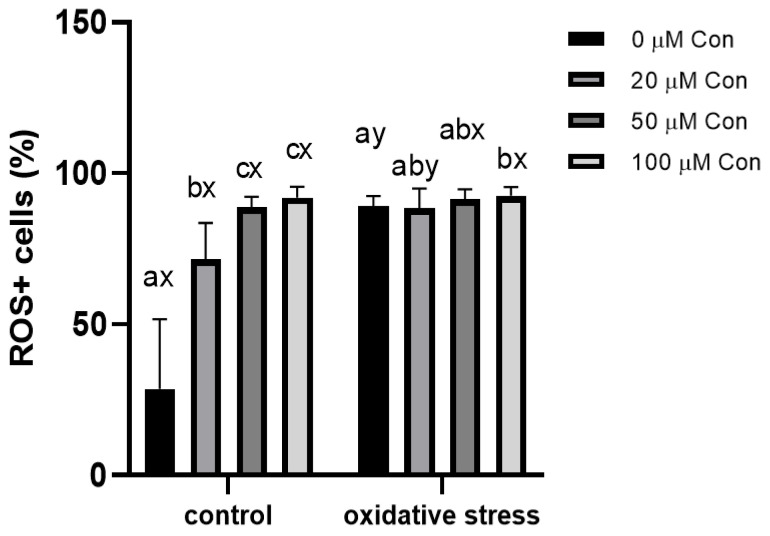
Effect of different concentrations of Con A on ROS-positive bull spermatozoa (%) incubated with different concentrations of Con A under control conditions (without menadione) and oxidative stress conditions (100 µM menadione). a, b, c: differences within control or oxidative stress between different concentrations of Con A. x, y: differences within a given concentration of Con A between control or oxidative stress (*n* = 6). Data are expressed as mean ± SEM; significance, *p* < 0.05.

The average population of ROS+ cells in the untreated sperm samples was 28.62 ± 21.06%, which increased in a concentration-dependent manner upon addition of Con A, up to 91.92 ± 3.28%. Incubation of sperm samples under menadione-induced oxidative stress caused an increase in ROS+ cell content, reaching 89.18 ± 3.03% of the cell population and increased to 92.52 ± 2.68% with the addition of Con A at 100 µM concentration (Figure 3).

### 2.4. Effect of Conoidin A on the Inhibition of Tyrosine Phosphorylation

**Figure 4:**
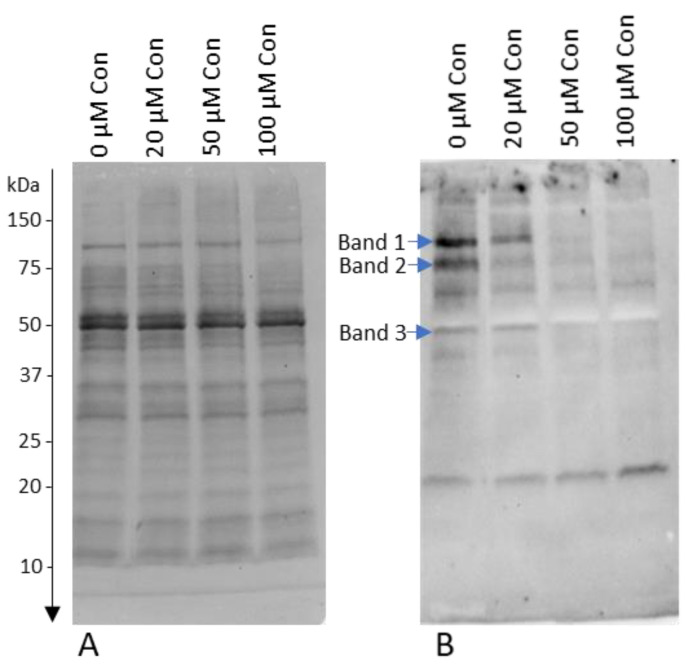
Influence of different concentrations of Con A on protein tyrosine phosphorylation. Western blot of tyrosine-phosphorylated proteins detected with anti-phosphotyrosine antibodies: (**A**) total proteins transferred to the membrane; and (**B**) marked bands of phosphorylated proteins were changed by the addition of Con A (*p* < 0.05).

Con A at a concentration of 100 µM caused the strongest inhibition of tyrosine phosphorylation, visible as a disappearance of bands with molecular weights of 109 (band 1), 83 (band 2), and 47 kDa (band 3), the intensity of which after adding 100 µM Con A decreased by 93.1%, 81.2%, and 57.0%, respectively, compared to 0 µM Con A (*p* < 0.05; Figure 4).

### 2.5. Identification of Proteins with Changed Levels of oxPTMs

**Figure 5:**
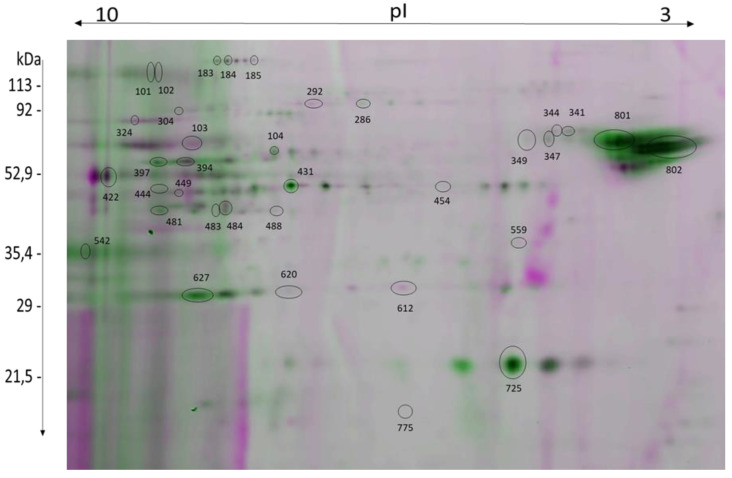
Representative 2D image of two superimposed gels showing oxPTMs of proteins extracted from bull sperm. Protein spots that are marked on the image show redox changes under different experimental conditions and are summarized and described in Table 1.

Using the gel-based redox proteomics approach, 35 protein spots showing differences in the level of oxPTMs under 2-Cys PRDX inhibition (with and without oxidative stress) and under the influence of oxidative stress (with and without 2-Cys PRDX inhibition) were detected and identified (Table 1, Figure 5, see supplementary Figure S1).

### 2.6. Effect of 2-Cys PRDX Inhibition on the Level of Protein oxPTMs

**Figure 6:**
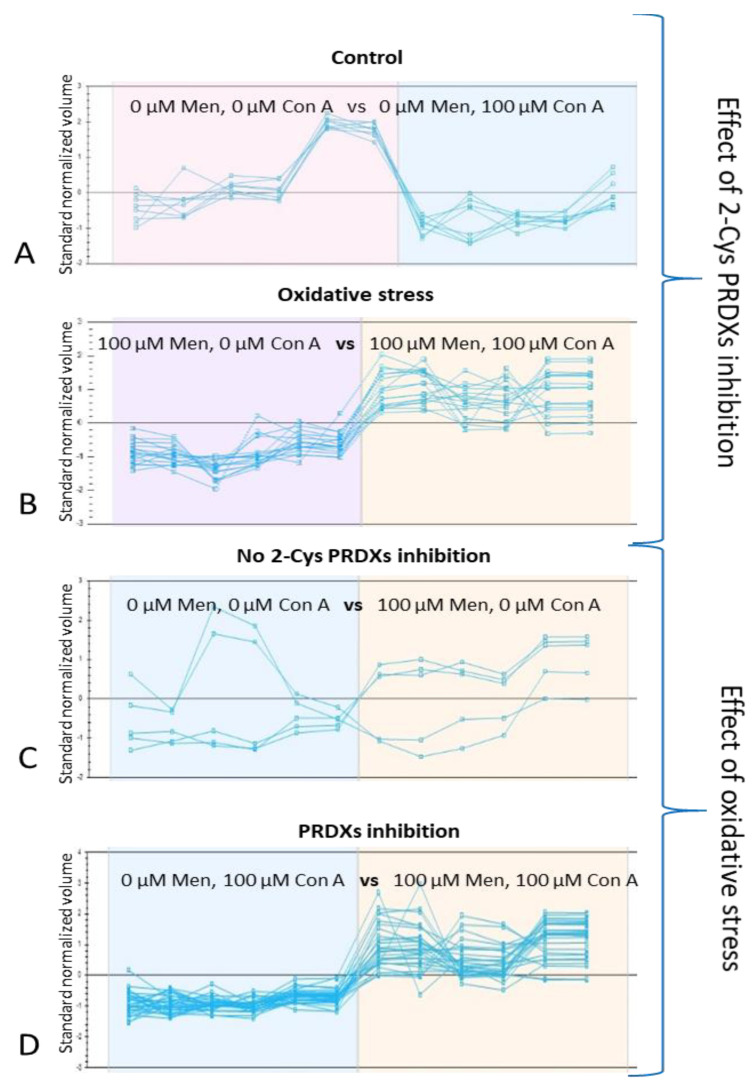
SameSpots software-generated graphs of identified bull sperm proteins, showing changes in fluorescence levels corresponding to the oxPTM level. Inhibition of 2-Cys PRDX under control conditions resulted in a decrease in the level of oxPTMs in 12 protein spots (**A**). Inhibition of 2-Cys PRDX under oxidative stress conditions caused an increase in the level of oxPTMs of 16 protein spots (**B**). The application of oxidative stress without 2-Cys PRDX inhibition resulted in a decrease in the level of oxPTMs of two protein spots and an increase in the level of oxPTMs of three protein spots (**C**). The application of oxidative stress with 2-Cys PRDX inhibition caused an increase in the level of oxPTMs of 16 protein spots (**D**).

Inhibition of 2-Cys PRDX (100 µM Con A) under control conditions (no oxidative stress) resulted in a decrease in the level of oxPTMs in 12 protein spots corresponding to nine proteins: LDHAL6B (spot 627), SDHA (spot 292), ACADM (spot 488), CS (spot 483), CCIN (spot 324), CAPZB (spot 612), ALDOA (spot 444), SPAM1 (spots 101, 102), and ACO2 (spots 183, 184, 185); see Figure 5 and Figure 6A.

**Figure 7:**
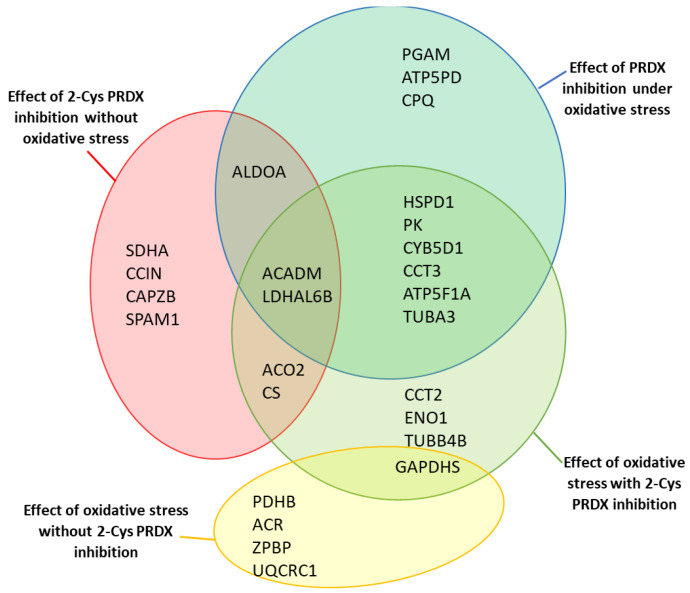
Diagrams showing the identified proteins that showed redox changes under different experi-mental conditions. Proteins with changed levels of oxPTMs due to 2-Cys PRDX inhibition without oxidative stress are marked by the red circle; proteins with changed levels of oxPTM due to 2-Cys PRDX inhibition under oxidative stress (100 µM menadione) are marked by the green circle; proteins with altered levels of oxPTM due to oxidative stress with inhibition of 2-Cys PRDX (100 µM Con A) are marked by the blue circle; proteins with altered levels of oxPTM due to oxidative stress without 2-Cys PRDX inhibition are marked by the yellow circle.

On the other hand, the effect was opposite when inhibition of 2-Cys PRDX was examined under oxidative stress (100 µM menadione). An increase in the level of oxPTMs was recorded in 16 protein spots (corresponding to 12 proteins): ACADM (spot 431), PGAM2 (spot 484), TUBA3 (spot 801), ATP5F1A (spots 397, 394), CCT3 (spot 286), CYB5D1 (spot 725), ATP5PD (spot 775), PK (spot 304), CPQ (spots 341, 349), HSPD1 (spots 344, 347), LDHAL6B (spots 620, 627), and ALDOA (spot 481); see Figure 5 and Figure 6B. Three common proteins (ALDOA, ACADM, and LDHAL6B) underwent changes in oxPTMs induced by the inhibition of PRDXs under both control and stress conditions (Figure 7).

### 2.7. Effect of Oxidative Stress on the Level of Protein oxPTMs

The application of oxidative stress (i.e., 100 µM menadione) without 2-Cys PRDX inhibition resulted in a decrease in the level of oxPTMs of two protein spots—PDHB (spot 559) and GAPDHS (spot 449)—and an increase in the level of oxPTMs of three protein spots—ACR (spot 422), ZPBP (spot 542), and UQCRC1 (spot 454); see Figure 5 and Figure 6C.

In turn, the application of oxidative stress with the simultaneous inhibition of 2-Cys PRDX (100 µM Con A) resulted in an increase in the level of oxPTMs in 15 protein spots (corresponding to 13 proteins): ACO2 (spot 185), PK (spots 304, 103), CCT2 (spot 104), CCT3 (spot 286), ATP5F1A (spots 394, 397), CS (spot 483), ACADM (spot 488), ENO1 (spot 431), LDHAL6B (spot 627), CYB5D1 (spot 725), HSPD1 (spot 347), TUBA3 (spot 801), and TUBB4B (spot 802); see Figure 5 and Figure 6D. Only one common protein (GAPDHS) was observed to undergo changes in oxPTMs induced by oxidative stress, both with and without PRDX inhibition (Figure 7).

## 3. Discussion

### 3.1. Research Assumptions and Rationale for Experimental Design

In our specific approach, we chose to inhibit the activity of 2-Cys PRDXs by a covalent inhibitor of 2-Cys PRDX activity: Con A [11]. To verify the effect of 2-Cys PRDX inhibition on sperm quality parameters, the motility, viability, and level of ROS+ cells were monitored at various Con A concentrations, both under control conditions and oxidative stress. In the next stage of the research, we investigated the effect of PRDX inhibition and the impact of oxidative stress on the oxPTM levels of sperm proteins.

### 3.2. Inhibition of 2-Cys PRDXs Affects ROS+ Spermatozoa Content

Sperm PRDXs are known primarily in terms of their well-established antioxidant functions [20]. Our results demonstrated that 2-Cys PRDXs are important for the protection of bull sperm from oxidative stress, as the inhibition of this group of enzymes increased the ROS+ cell number under control conditions in a dose-dependent manner. The results obtained by [11] also showed that the percentage of ROS+ cells in human sperm (measured with Mitosox-red) increased with the addition of Con A, but a significant increase in the percentage of ROS+ cells was observed at a higher concentration of Con A (80 µm) than for the bull sperm in our study (20 µm). This may indicate 2-Cys PRDX has greater antioxidant importance in bull sperm compared to human sperm.

Under oxidative stress conditions, there was no significant difference in ROS+ sperm with increasing Con A dose according to the cytometric analysis, but the differences were evident at the level of protein oxidation (see below). In addition, it is well-known that, under conditions of strong oxidative stress, PRDXs undergo hyperoxidation, which is associated with the reduction in their peroxidative activity and gaining the molecular functions of chaperones [14].

### 3.3. Inhibition of 2-Cys PRDXs Affects Sperm Motility without Changing Viability

Our results showed that Con A, even at the lowest concentration used, drastically reduced sperm motility under both control and oxidative stress conditions. A strong inhibitory effect on motility has also been observed in mouse sperm under the influence of Con A at a dose as low as 10 μM [20], which suggests that 2-Cys PRDXs are significantly involved in sperm movement in different mammalian species. Surprisingly, in our study, the loss of sperm motility was not accompanied by a decrease in viability, which suggests that inhibition of 2-Cys PRDXs affects motility/motion kinematics, but does not appear to be toxic to spermatozoa, as has also been recorded for mouse sperm by [20].

It is not known why the inhibition of 2-Cys PRDXs causes such a drastic decrease in sperm motility without significantly affecting their viability. Most likely, it is caused by the accumulation of H_2_O_2_, but also a decrease in ATP with the simultaneous inhibition of protein tyrosine phosphorylation [20], which was also confirmed in our research. This suggests that the mechanism responsible for the decrease in sperm motility by 2-Cys PRDX inhibition may be related to exhaustion of energy and phosphorylation disorders crucial for sperm movement.

### 3.4. Inhibition of PRDX Activity Results in Opposite Changes in the Level of Protein oxPTMs Depending on the Absence or Presence of Oxidative Stress

**Figure 8:**
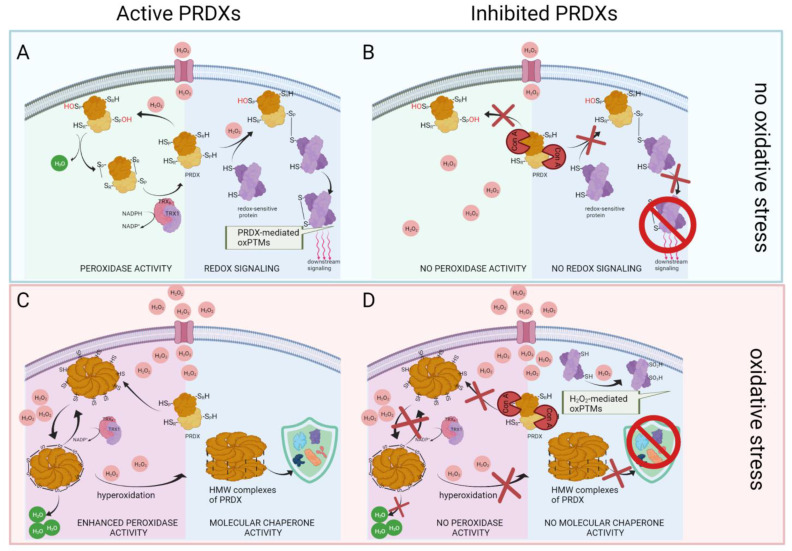
Theoretical image of the effects of inhibition of PRDX activity under non-stress and oxidative stress conditions. In non-stress conditions, active 2-Cys PRDX scavenges H2O2 through the formation of a sulfenic acid (Cys-SOH) on the peroxidatic cysteine (CysP), with the subsequent formation of a disulfide bond between Cysp and the resolving cysteine (CysR) [32]. Oxidative equivalents can be transferred from PRDX to redox-sensitive target proteins, thus enabling re-dox-signaling [13] (**A**). Covalent binding of Con A to the peroxidatic cysteines of 2-Cys PRDXs results in the inhibition of peroxidative and redox relay activity [24] (**B**). Under oxidative stress conditions, an equilibrium exists between the dimer and decamer structures, with the reduced (SH) decamer being the most efficient peroxidase [13]. The decamers can further undergo overoxidation and form high-molecular-weight (HMW) oligomers exhibiting molecular chaperone functions [14] (**C**). Inhibition of 2-Cys PRDX under oxidative stress conditions results in lack of both the peroxidative function of PRDX and the molecular chaperone function (**D**).

Peroxiredoxins are excellent H_2_O_2_ sensors [13], and perform various functions depending on whether the peroxide concentration in the cell is at the physiological level (Figure 8A) or has reached a level indicative of oxidative stress [14] (Figure 8C). Our results also strongly suggest the multifunctionality of 2-Cys PRDXs, as the inhibition of PRDXs caused a decrease or increase in sperm protein oxPTMs, depending on the oxidative circumstances.

At physiological concentrations of H_2_O_2_, 2-Cys PRDXs act as sensors and transducers of redox signaling [25,26]. Inhibition of 2-Cys PRDX activity without oxidative stress caused a decrease in oxPTMs in all detected sperm proteins, which suggests that, under physiological conditions, 2-Cys PRDXs are involved in redox signaling through the use of reversible oxidative modifications of a specific group of redox-sensitive proteins (Figure 8B). Such direct redox interactions between 2-Cys PRDXs and redox-sensitive target proteins occur in several signaling pathways, such as apoptosis signaling [27,28], cytokine-induced STAT3 signaling [9], platelet-derived growth factor signaling [29], MAP kinase signaling [30], and replisome activity regulation [31] of somatic cells. In sperm, the role of 2-Cys PRDXs in homeostatic signaling is poorly understood. There are strong indications of the involvement of PRDXs in redox signal transduction mediating sperm capacitation [23]; however, unlike in somatic cells, the mechanism of interaction with specific partner proteins is not known. In view of the knowledge obtained in our study, it is desirable to conduct further experiments aimed at identifying and characterizing signaling pathways related to PRDXs in spermatozoa.

On the other hand, in the presence of oxidative stress, 2-Cys PRDXs oligomerize into decameric structures and further undergo hyperoxidation, forming high-molecular-weight structures acting as molecular chaperones [13,14] (Figure 8C). In our study, inhibition of 2-Cys PRDX activity under oxidative stress resulted in an increase in protein oxPTM levels, suggesting that the detected oxidative modifications resulted from direct oxidation due to elevated peroxide concentration, which was also indicated by an increase in the level of ROS+ cells. There is an equally possible explanation concerning the impossibility of inhibited PRDXs fulfilling the chaperone function [15] (Figure 8D). To confirm these assumptions, further studies are needed to investigate the polymerization status of 2-Cys PRDX under oxidative stress with or without Con A.

### 3.5. Inhibition of 2-Cys PRDXs in Non-Oxidative Conditions Leads to Decrease in Sperm Protein oxPTMs

The current knowledge on the role of PRDXs in non-stress conditions indicates that, in addition to maintaining low concentrations of H_2_O_2_, PRDXs are involved in redox signal transduction [9]. Oxidative equivalents can be transferred from H_2_O_2_ to peroxiredoxins, and further to the target proteins, by thiol–disulfide exchange [13]. Such redox interactions between peroxiredoxins and STAT3 [9], ASK1 [27], and TIMELESS [31] have been extensively studied in human somatic cell cultures. In sperm cells, PRDXs play important roles, not only as antioxidants but, first and foremost, as key regulatory elements in the physiology of fertilization and for redox signal transmission during capacitation [23]. The relationship between PRDXs and capacitation has also been detailed by [16], showing that inhibitors of peroxiredoxins prevent sperm capacitation and actin polymerization without altering sperm viability.

In the current study, we observed a decrease in protein oxPTMs caused by the addition of Con A, which suggests that, under non-stress conditions, the group of sperm proteins is oxidatively modified due to the action of 2-Cys PRDXs. Interestingly, these proteins are of mitochondrial (LDHAL6B, CS, ACO2, SDHA, ACAPM) and actin cytoskeleton origin (CAPZB, ALDOA, CCIN) and are closely related to energy metabolism and motility. As the inhibition of 2-Cys PRDX under non-stress conditions also induced a significant reduction in motility, it is possible that the oxidative modifications of the mitochondrial and cytoskeleton proteins, provided by the activity of 2-Cys PRDXs, are an important element of the sperm motility mechanism. As 2-Cys PRDX-mediated transfer of oxidative equivalents may regulate sub-cellular localization, structural conformation, and functionality of target proteins [13], disturbances of sperm motility may be caused directly by the lack of appropriate oxidative modifications that assure the physiological properties of redox-sensitive proteins.

### 3.6. Inhibition of 2-Cys PRDXs in Non-Oxidative Conditions Leads to a Decrease in Sperm Protein Tyrosine Phosphorylation

At present, it is widely held that tyrosine phosphorylation of sperm proteins is a redox-regulated process [33,34,35]. Important sperm functions, including activated motility, may also be redox regulated [36]. As our study revealed that 2-Cys PRDX inhibition decreased protein phosphorylation (Figure 4), the possibility of signaling dysregulation relates to the close relationship between 2-Cys PRDX-mediated redox signaling and phosphorylation events [28,37]. Our results suggest that the inhibition of 2-Cys PRDX interferes with PRDX-mediated oxidative modification of the sperm proteins, preventing the sequential phosphorylation cascade from being triggered; in turn, the lack of proper phosphorylation of sperm proteins leads to a loss of sperm motility [36,38]. Our results indicated that the inhibition of 2-Cys PRDX under physiological conditions, in addition to a strong decline of motility, also inhibits protein phosphorylation, in agreement with the study of [20]. Further research should be conducted to identify proteins whose phosphorylation decreases with 2-Cys PRDX inhibition in order to unravel the mechanism of the relationship between redox signaling (modulated by 2-Cys PRDXs) and the protein phosphorylation cascade.

### 3.7. Oxidative Stress Causes Oxidation of Glycolytic Enzymes That Intensifies after Inhibition of PRDXs

Sperm rate-limiting glycolytic enzymes (PKM and GAPDH) possess numerous redox-sensitive Cys, and are known to be inactivated by oxidation [39]. Our results revealed that the oxidation of PKM and GAPDHS (sperm-specific isoform of GAPDH) under oxidative stress was dependent on whether 2-Cys PRDX was active or inhibited (Figure 7). Oxidative stress in sperm with active PRDX caused GAPDHS oxidation, while both glycolytic enzymes PKM and GAPDHS were oxidized with inhibited PRDX. Additionally, the oxidation of other glycolytic enzymes—ENO1 and LDHAL6B—was also observed along with inhibited 2-Cys PRDX. The oxidation of glycolytic enzymes did not take place under the influence of PRDX inhibition in the absence of oxidative stress, which confirms that the oxidation of glycolytic sperm enzymes is a defense reaction to oxidative stress [40]. The oxidation of PKM and GAPDHS detected in our study has been widely recognized as a metabolic switch from glycolysis to the pentose phosphate pathway (PPP) in both somatic cells [39,41,42] and spermatozoa [40,43,44]. Under oxidative stress conditions, spermatozoa switch glucose metabolism from glycolysis to PPP to provide the cell with NADPH, which is the main function of this pathway in spermatozoa [45]. NADPH serves as a source of reducing equivalents in the enzymatic removal of H_2_O_2_, which is crucial for the reactivation of glutaredoxins and PRDXs [39]. Activation of the PPP pathway by oxidation of rate-limiting glycolytic enzymes has been recognized as a protective mechanism against oxidative stress in human [46] and boar sperm [40].

In summary, our results indicated that the presence of oxidative stress, even with active PRDX, inhibits glycolysis by oxidation of the glycolytic enzyme GAPDHS. Simultaneous inhibition of PRDX enhanced this effect, resulting in the oxidation of other glycolytic proteins: PKM, ENO1, and LDHAL6B (Figure 7). This may result in a switching of glucose metabolism from glycolysis to PPP—the sperm′s defense strategy against oxidative stress. The inhibition of glycolysis results in a decrease in the production of ATP, which is necessary for the maintenance of sperm motility and phosphorylation, as demonstrated in our research.

### 3.8. Inhibition of 2-Cys PRDXs under Oxidative Stress Conditions Leads to an Increase in Sperm Protein oxPTM Level

Our results showed that, under oxidative stress, many more proteins were oxidized after the inhibition of 2-Cys PRDXs (14 protein spots) than without blocking the activity of this enzyme (three protein spots). The oxidized proteins were identified as glycolytic enzymes (GAPDHS, PKM, ENO1, LDHA6B), TCA pathway enzymes (ACO2, CS), respiratory chain enzymes (CYB5D1, ATP5F1A, ACADM), chaperones (CCT2, CCT3, HSPD1), and sperm axonemal tubulins (TUBA3, TUBB4B). The increased level of oxPTMs in such a large quantity of sperm proteins may be due to two reasons. First, under the influence of oxidative stress, blocked 2-Cys PRDXs do not have peroxidase activity, leading to the accumulation of large amounts of H_2_O_2_ and, consequently, to H_2_O_2_-mediated oxidation of proteins (oxidative damage of proteins; see Figure 8D). Secondly, the inhibition of PRDXs by covalent bonding of Con A prevents the formation of high-molecular-weight complexes, which arise under the influence of oxidative stress with active 2-Cys PRDX and act as molecular chaperones [14]. A lack of the chaperone function of PRDXs means that the protein conformation is not protected against the harmful effects of free radicals, leading to further oxidative damage of sperm proteins.

## 4. Materials and Methods

Unless otherwise stated, all chemicals were purchased from Sigma-Aldrich (St. Louis, MO, USA).

### 4.1. Research Material

Fresh semen was collected, by the employees of the Mazowieckie Center for Animal Breeding and Reproduction (Łowicz, Poland), from six sexually mature Holstein Friesian bulls (*n* = 6). The semen was diluted 1:1 in protein-free Bioxcel diluent (IMV Technologies, L′Aigle, France), transported to the laboratory at 4 °C within 3 h of harvesting, and stored at 15 °C for 12 h at most.

### 4.2. Sperm Preparation

Prior to the analyses, the spermatozoa were separated from the diluent by centrifugation at 300× *g* for 20 min, washed with BO-SemenPrep medium (IVF Bioscience, Falmouth, UK), and centrifuged at 500× *g* for 5 min. The obtained sperm pellet was resuspended in a suspension buffer containing 100 mM NaCl, 3.1 mM KCl, 2 mM CaCl_2,_ 0.3 mM Na_2_HPO_4_, 21.6 mM Na-Lactate, 0.4 mM MgCl_2_ × 4H_2_O, 25 mM NaHCO_3_, 10 mM Hepes, and 1 mM Na-pyruvate to a final sperm concentration of 100 million/mL.

### 4.3. 2-Cys PRDX Inhibition under Control or Oxidative Stress Conditions

2-Cys PRDX activity in bovine sperm preparations was inhibited by the addition of Conoidin A (Millipore, Billerica, MA, USA), according to procedure described by [20], to different final concentrations (0, 20, 50, and 100 µM). Previously, a 50 mM stock of Con A was prepared in dimethyl sulfoxide (DMSO) and stored at −20 °C for up to two weeks. Sperm samples were incubated with Conoidin A for 30 min at 38 °C with 5% CO_2_, in a humidified atmosphere. Then, the sperm samples were incubated for another 30 min with suspension buffer or suspension buffer supplemented with 100 µM menadione (oxidizing agent).

### 4.4. Sperm Motility and Viability Measurement

The motility of each sperm sample was assessed at the end of the incubation period using a computer-assisted sperm analysis system (CASA, CEROS II system, Hamilton-Thorne, Beverly, MA, USA). Sperm viability was measured using a Count and Viability Kit (Luminex, Austin, TX, USA), according to the manufacturer′s instructions, with a GUAVA easyCyte 8HT Benchtop Flow Cytometer (Guava Technologies Inc., Luminex, Austin, TX, USA). The InCyte guavaSoft™ 4.0 software was used for data acquisition and analysis.

### 4.5. Sperm Oxidative Status Evaluation

The CellROX Green fluorescent reagent (Molecular Probes, Eugene, OR, USA) was used to assess the oxidative status of bovine sperm cells under the applied experimental conditions. The reagent was added, for a final concentration of 6.25 μM, to aliquots containing 20 × 10^6^ cells/mL and incubated for 30 min at 38 °C in a 5% CO_2_ humidified atmosphere. CellROX Green-labeled samples were excited with a blue 488 nm laser, and the emitted fluorescence was captured by means of a 515/30 nm GREEN-B filter set with a GUAVA easyCyte 8HT Benchtop Flow Cytometer (Guava Technologies, Hayward, CA, USA). The InCyte guavaSoft™ 4.0 software was used for data acquisition and analysis. The control ROS-positive (ROS+) sperm cell population was obtained by adding 2.5 mM menadione to a representative sperm sample and the ROS-negative (ROS−) cell population was determined using 3 mM of the antioxidant N-acetylcysteine.

### 4.6. Protein Extraction

To extract proteins, 24 sperm samples were centrifuged at 900× *g*/10 min/4 °C, washed with phosphate-buffered saline (PBS), resuspended in a lysis buffer (7 M urea, 2 M thiourea, 4% (*w*/*v*) 3-((3-cholamidopropyl)-dimethylammonio)-1-propanesulfonate (CHAPS), 2.5% (*v*/*v*) protease inhibitor cocktail (firma), 1% (*v*/*v*) phosphate inhibitor cocktail, and 0.1 mM neocuproine) and sonicated on ice three times for 7 s at 30% amplitude using a VCX-130 Ultrasonic Processor (Sonics & Materials, Inc., Newtown, CT, USA). To inhibit the thiol–disulfide exchange reaction, samples were precipitated with 10% trichloroacetic acid (TCA), washed twice with cold acetone to remove acid, and resuspended in rehydration buffer (7 M urea, 2 M thiourea, and 4% CHAPS).

### 4.7. Blocking Free Thiols and Labeling Oxidized Cysteines

Blocking of protein-free thiols and labeling of oxidized cysteines were performed, according to the manufacturer′s instructions, with the reagents included in the Saturn-2D REDOX Labeling Kit (DyeAGNOSTICS, Halle, Germany). Briefly, 5 µg of each protein sample was mixed with 4 µL of redox labeling buffer and 3 µL of cysteine interacting compound (CinC) then incubated at 35 °C for 1 h in order to block all the reduced cysteines and prevent their further labeling. Then, 3 µL of redox stop solution was added for an additional 10 min, and the excess CinC was removed using the spin columns included in the Saturn-2D REDOX Labeling Kit. Reduction in the remaining oxidized cysteines was carried out by incubation with 2.5 µL tris(2-carboxyethyl)phosphine (TCEP) for 1 h at 35 °C. Cysteines reduced as such were labeled with 5 µL of S-Dye300. After 1 h at 35 °C, the labeling reaction was stopped with 6 µL redox stop solution for 10 min. Prior to gel electrophoresis, each sample was mixed with the same quantity of the internal standard of proteins and run simultaneously on a single gel. An internal protein standard was prepared analogously by labeling the pool of all the samples from the experiment with a maleimide-based dye (S-Dye200), which served as further reference to allow gel–gel normalization.

### 4.8. D-PAGE Separation of Fluorescently Labeled Proteins

Five µg of each protein sample (labeled as described above) was mixed with the same quantity of internal standard proteins and loaded onto 13 cm IPG Immobiline DryStrips with a non-linear 3–10 pH gradient (GE Healthcare, Chicago, IL, USA). The IPG strips were passively rehydrated for 18 h prior to protein isoelectric focusing, and separation was conducted using the Ettan IPGphor apparatus (GE Healthcare, Chicago, IL, USA). The following IEF program was used: 500 V for 1 h (step and hold), 1000 V for 1 h (gradient), 8000 V for 2.5 h (gradient), and 8000 V for 0.35 h (step and hold), with a total focusing time of 16.8 kVh and current limited to 50 μA per IPG strip. After isoelectric focusing, the IPG strips were incubated for 15 min in equilibration buffer (6 M urea, 75 mM Tris-HCl (pH 8.8), 29.3% glycerol, 2% SDS, and trace bromophenol blue) supplemented with 10 mg/mL DTT, then for another 15 min in SDS equilibration buffer supplemented with 25 mg/mL iodoacetamide. The equilibrated strips were then transferred to 12.5% polyacrylamide gels for a second dimension of 2D electrophoresis and separated at 1 W/gel in an Ettan Dalt-Six apparatus (GE Healthcare, Chicago, IL, USA) for 16 h.

### 4.9. Image Acquisition and Identification of Proteins

The 2D gels were scanned using a Typhoon 9500 FLA scanner (GE Healthcare, Chicago, IL, USA) at excitation/emission wavelengths of 532/576 (S-Dye200) and 633/664 nm (S-Dye300), respectively. The SameSpots software 5.1.012 (TotalLab, Newcastle upon Tyne, UK) was used to detect, normalize, and quantify the obtained spot patterns. Differences in the fluorescence intensity of protein spots, reflecting the degree of cysteine oxidation, were analyzed using Student′s *t*-test analysis included in the software. Proteins for which the intensity of fluorescence changed between experimental conditions (*p* < 0.05) were selected for further mass spectrometry identification. The selected spots were excised from the additional preparative gels, which were loaded with 200 μg of proteins prepared accordingly, but without fluorescent labeling. To allow for spot excision, proteins on the gel were stained with Coomassie brilliant blue dye and identified using matrix-assisted laser desorption/ionization time-of-flight/time-of-flight (MALDI-TOF/TOF) mass spectrometry (MS), as described previously [47]. Mascot search parameters were as follows: trypsin cleavage, one missed cleavage site allowed, oxidation of methionine as the variable modification, carbamidomethylation of cysteine as the fixed modification, fragment ion mass tolerance of 0.2 Da, and parent ion mass tolerance of 50 ppm. The identification result was regarded as successful when at least two of the identified peptides per protein had an ion score ≥30 with the ion mass tolerance of 0.7 Da and parent ion mass tolerance of 100 ppm.

### 4.10. Evaluation of Tyrosine Phosphorylation of Bull Sperm

Sperm samples were washed 3× by centrifugation (350× *g* for 10 min at room temperature) with PBS buffer supplemented with 1 mM sodium orthovanadate (tyrosine phosphatase inhibitor). Sperm pellets were suspended in Laemmli buffer (31.25 mM Tris-HCl pH 6.8, 1.275% SDS, 5% glicerol, 2.5% 2-mercaptoethanol, 0.002% bromophenol blue), boiled for 10 min, and centrifuged at 14,000× *g* for 10 min to remove cell debris. Proteins were resolved by SDS-PAGE electrophoresis using 12% Mini-PROTEAN TGX Stain-Free Precast Gels (Bio-Rad, Hercules, CA, USA). After electrophoresis, the stain-free gels were activated for 45 s and scanned in the ChemiDoc Touch Imaging System (Bio-Rad, Hercules, CA, USA). Proteins from the gel were then transferred to nitrocellulose membranes and scanned again to obtain a stain-free image of all the transferred proteins. The membranes were blocked overnight in a cold room with 2% skim milk dissolved in Tris-Buffered Saline with 0.1% Tween 20 (TBST). Tyrosine phosphorylation of the proteins was detected with the Western blot technique using primary antibodies 4G10 Platinum (Sigma-Aldrich. St. Louis, MO, USA) diluted 1:2000 in TBST and HRP-conjugated Immun-Star anti-mouse secondary antibodies diluted 1:25,000 in TBST (Bio-Rad, Hercules, CA, USA), and visualized using Clarity Western ECL Substrate (Bio-Rad). The stain-free blot image of total proteins, captured after Western blot, was used as a loading control.

### 4.11. Statistical Analyses

The results are presented as the mean ± standard error of the mean (SEM) (*n* = 6). All analyses were performed at a significance level of *p* < 0.05 using the GraphPad Prism software v6.02 (GraphPad Software Inc. San Diego, CA, USA). Data expressed as a percentage (sperm motility, viability, ROS+ cells) were normalized by arcsine square root transformation. Data were analyzed using two-way repeated measures ANOVA, followed by Tukey’s test for post hoc comparison of means. To analyze protein gels, the statistical component of the SameSpots software was used, as described above.

## 5. Conclusions

The inhibition of 2-Cys PRDX activity may result in an increase or decrease in oxPTMs in bull sperm proteins, depending on the oxidative circumstances. Inhibition of 2-Cys PRDX activity in the absence of oxidative stress caused a decrease in the level of oxPTMs of specific mitochondrial and actin cytoskeleton proteins closely related to the energy metabolism and motility of the sperm, suggesting the existence of 2-Cys PRDX-dependent redox regulation under physiological conditions. It is possible that the oxPTMs of the mitochondrial and cytoskeletal proteins provided by the activity of 2-Cys PRDXs are important elements of the sperm motility mechanism and that disturbances to sperm motility may be caused directly by a lack of appropriate PRDX-mediated oxPTMs, which ensure the physiological properties of redox-sensitive proteins. Under oxidative stress, many more proteins were oxidized after PRDX inhibition (14 protein spots) than without blocking the activity of this enzyme (three protein spots), suggesting that, under oxidative stress conditions, 2-Cys PRDX activity is focused on antioxidant activity, protecting glycolytic, TCA pathway, and respiratory chain enzymes; chaperones; and sperm axonemal tubulins from oxidative damage. Interestingly, the inhibition of PRDX resulted in oxidation of a group of rate-limiting glycolytic proteins, which is known to trigger the switching of glucose metabolism from glycolysis to PPP. Therefore, the mechanism responsible for the decrease in sperm motility by 2-Cys PRDX inhibition may be related to a decrease in the production of ATP necessary for the maintenance of sperm motility and phosphorylation, which was confirmed in our research.

## Figures and Tables

**Table 1:** Identified proteins showing changes in oxPTMs caused by inhibition of 2-Cys PRDXs with Con A and/or oxidative stress.

Spot No.	Protein Name	Gene Name	Accession Number	Calculated MW/pI	Protein Score/Number of Peptides	No. of Cysteines
101	Hyaluronidase	SPAM1	F1MTV1 NP_001008413.3	63268/8.79	244/2	18
102	Hyaluronidase	SPAM1	F1MTV1 NP_001008413.3	63268/8.79	353/3	18
103	Pyruvate kinase	PKM	A5D984	58482/7.96	215/2	10
104	T-complex protein 1 subunit beta	CCT2	Q32BHO	57781/6.18	187/2	6
183	Aconitate hydratase, mitochondrial	ACO2	A0A3Q1M6K6 XP_005909496.1	85481/7.67	164/1	13
184	Aconitate hydratase, mitochondrial	ACO2	A0A3Q1M6K6 XP_005909496.1	85481/7.67	164/1	13
185	Aconitate hydratase, mitochondrial	ACO2	P20004 XP_005909496.1	86047/7.87	244/1	13
286	T-complex protein 1 subunit gamma	CCT3	Q3T0K2 ELR54279.1	61118/6.38	470/3	10
292	Succinate dehydrogenase (ubiquinone) flavoprotein subunit, mitochondrial	SDHA	G3MY67 XP_027375926.1	74101/7.55	906/10	19
304	Pyruvate kinase	PK	A5D984	58482/7.96	220/2	10
324	Calicin	CCIN	Q28068 XP_005908650.1	67873/8.49	414/4	18
341	Carboxypeptidase Q	CPQ	A0A3Q1LSW9 XP_005215732.1	52902/5.83	356/3	1
344	60 kDa chaperonin	HSPD1	F1MUZ9 XP_015313986.1	61110/5.71	330/4	3
347	60 kDa chaperonin	HSPD1	F1MUZ9 XP_015313986.1	61110/5.71	1010/7	3
349	Carboxypeptidase Q	CPQ	A0A3Q1MFF3	49530/5.52	188/2	5
394	ATP synthase subunit alpha	ATP5F1A	F1MLB8 XP_019842301.1	59767/9.21	399/2	2
397	ATP synthase subunit alpha	ATP5F1A	F1MLB8 XP_019842301.1	59767/9.21	496/4	2
422	Acrosin	ACR	G5E5C6	46149/9.64	185/2	12
431	Alpha-enolase	ENO1	Q9XSJ4 NP_776474.2	47639/6.37	291/3	6
444	Fructose-bisphosphate aldolase	ALDOA	A6QLL8	39925/8.45	223/2	9
449	Glyceraldehyde-3-phosphate dehydrogenase, testis-specific	GAPDHS	Q2KJE5	43659/8.32	152/2	5
454	Cytochrome b-c1 complex subunit 1, mitochondrial	UQCRC1	P13272	29813/9.04	393/3	5
481	Fructose-bisphosphate aldolase	ALDOA	A6QLL8 XP_006070551.2	39925/8.45	283/3	9
483	Citrate synthase, mitochondrial	CS	Q29RK1	51968/8.16	378/4	4
484	Phosphoglycerate mutase 2	PGAM2	Q32KV0 NP_001033200.1	28838/8.99	203/2	3
488	Medium-chain specific acyl-CoA dehydrogenase, mitochondrial	ACADM	Q3SZB4 NP_001068703.1	46943/8.31	505/4	7
542	Zona pellucida binding protein	ZPBP	F1N369	40313/9.14	312/2	15
559	Pyruvate dehydrogenase E1 component subunit beta, mitochondrial	PDHB	P11966	39443/6.21	271/3	6
612	F-actin-capping protein subunit beta	CAPZB	A0A3Q1MG13 XP_005203140.1	34782/5.59	336/2	7
620	L-lactate dehydrogenase A-like 6B	LDHAL6B	Q3T056	42022/8.91	379/4	8
627	L-lactate dehydrogenase A-like 6B	LDHAL6B	Q3T056 NP_001030352.1	42022/8.91	379/4	8
725	Cytochrome b5 heme-binding domain-containing protein	CYB5D1	A0A3Q1LQK0	26704/5.36	194/3	1
775	ATP synthase subunit d, mitochondrial	ATP5PD	P13620	18738/5.99	436/5	1
801	Tubulin alpha-3 chain isoform X3	TUBA3	XP_024833647.1	46605/4.99	828/7	9
802	Tubulin beta-4B chain	TUBB4B	NP_001029835.1	50255/4.79	498/6	8

## Data Availability

All data underlying this article are available directly in the article text.

## Supplementary Materials

The following are available online at https://www.mdpi.com/article/10.3390/ijms222312888/s1.
