# Supplementary material for: Effect of 2-Cys Peroxiredoxins Inhibition on Redox Modifications of Bull Sperm Proteins
Source: Int J Mol Sci. 2021 Nov 28;22(23):12888. doi: 10.3390/ijms222312888 (PMC8657687; doi:10.3390/ijms222312888)
Supplement: Supplementary file 1 [file ijms-22-12888-s001.zip › ijms-1456279-SI.pdf]

Supplementary data 1

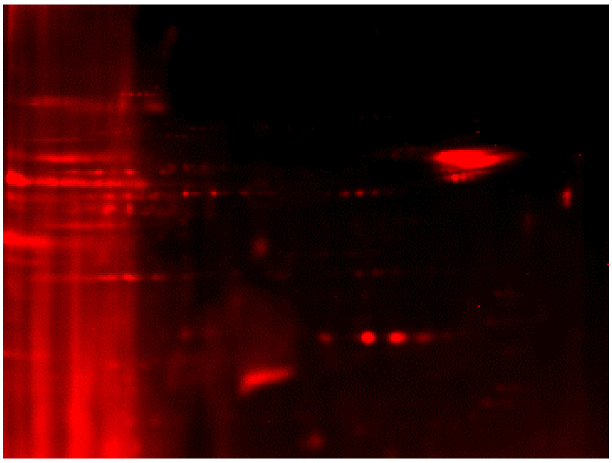

S-dye 300 (Sample Men0Con0)

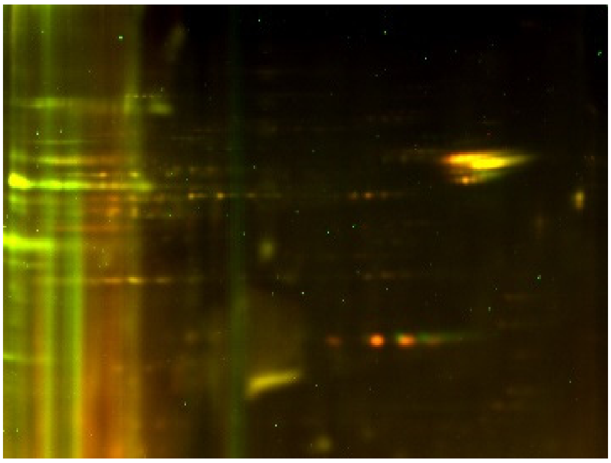

Overlay of S-dye 300 and S-dye200

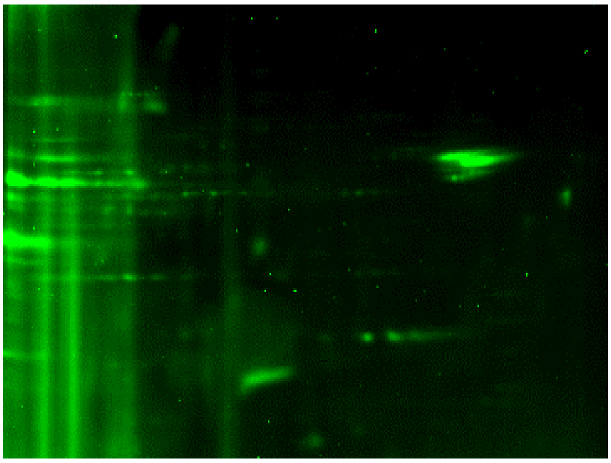

S-dye 200 (internal standard)

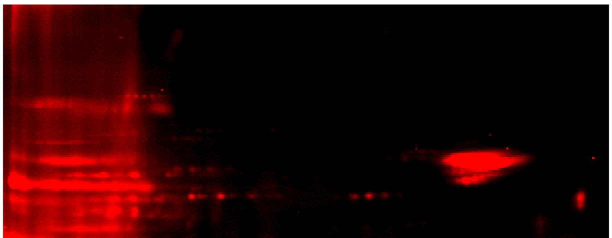

S-dye 300 (Sample Men100Con0)

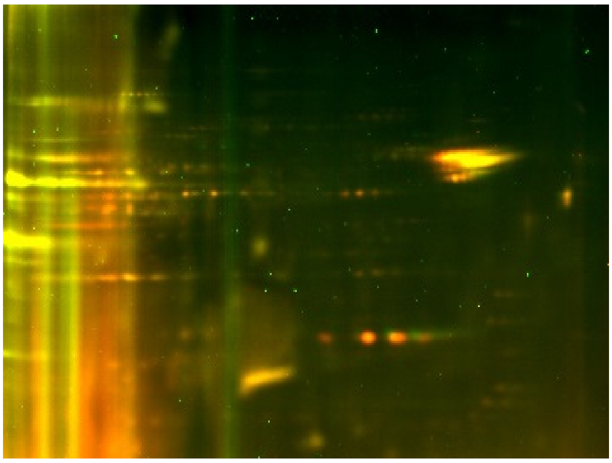

Overlay of S-dye 300 and S-dye200

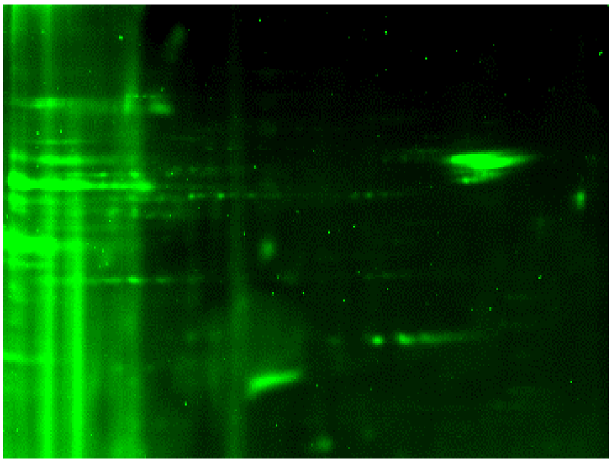

S-dye 200 (internal standard)

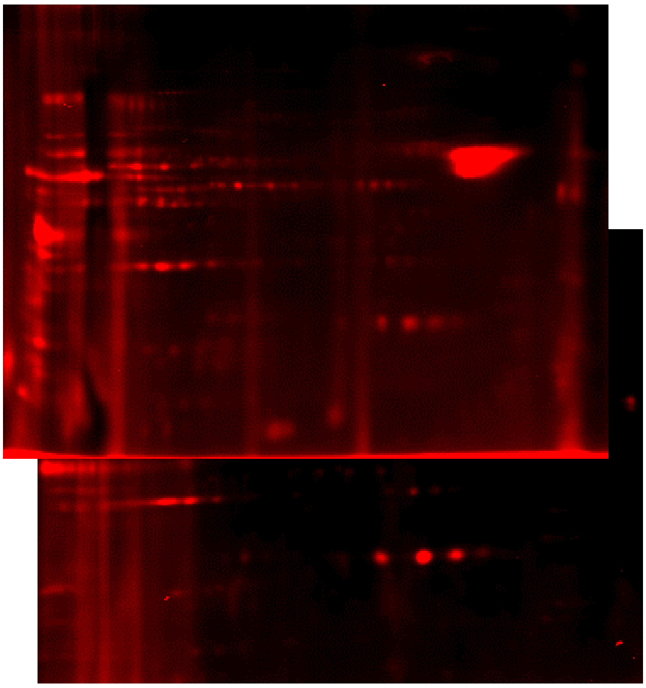

S-dye 300 (Sample Men0Con100)

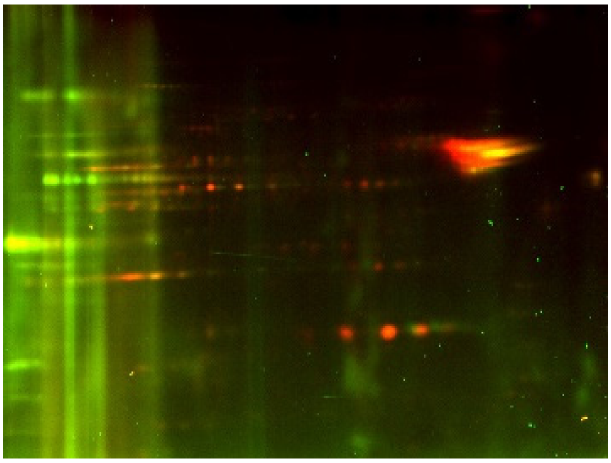

Overlay of S-dye 300 and S-dye200

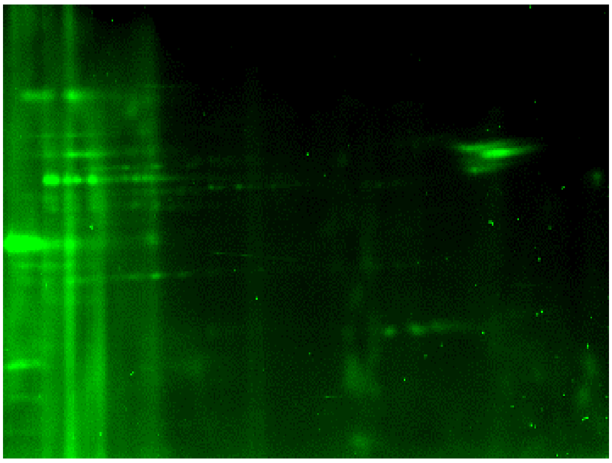

S-dye 200 (internal standard)

S-dye 300 (Sample Men100Con100)

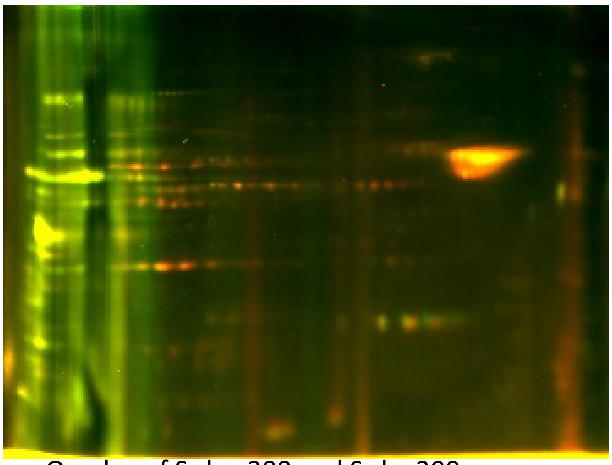

Overlay of S-dye 300 and S-dye200

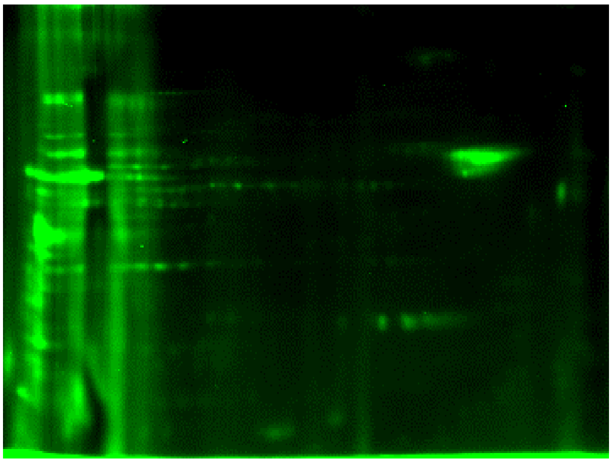

S-dye 200 (internal standard)
